# Antimicrobial Consumption and Utilisation in Zambia: Results from the Analysis of National Data for the Human and Animal Health Sectors

**DOI:** 10.3390/antibiotics14111126

**Published:** 2025-11-07

**Authors:** Steward Mudenda, Joseph Yamweka Chizimu, Victor Daka, Maisa Kasanga, Webrod Mufwambi, Kelvin Mwangilwa, Jimmy Hangoma, Priscilla Gardner, Chikwanda Chileshe, Ntombi B. Mudenda, Taona Sinyawa, Amon Siame, Mwendalubi Hadunka, Fred Mulako Simwinji, Kaunda Kaunda, Mpela Chibi, Zoran Muhimba, Elimas Jere, Makomani Siyanga, Peter Lisulo, Freddie Masaninga, Samson Mukale, Andrew Bambala, Misheck Shawa, Charles Chileshe, Bertha Chibwe, Mercy Mukuma, Bruno S. J. Phiri, Geoffrey Mainda, Yasuhiko Suzuki, Fusya Goma, John Bwalya Muma, Roma Chilengi

**Affiliations:** 1 Zambia National Public Health Institute, Antimicrobial Resistance Coordinating Committee, Lusaka 10101, Zambia; anita.kasanga@znphi.gov.zm (M.K.); mwangilwakelvin@yahoo.com (K.M.); priscillagardner82@gmail.com (P.G.); zmuhimba@yahoo.com (Z.M.); mukalesamson14@gmail.com (S.M.); chichalesi2@gmail.com (C.C.);; 2 Department of Pharmacy, School of Health Sciences, University of Zambia, Lusaka 10101, Zambia; webrod.mufwambi@unza.zm; 3 Public Health Department, School of Medicine, Copperbelt University, Ndola 10101, Zambia; dakavictorm@gmail.com; 4 University Teaching Hospital, Lusaka 10101, Zambia; bambalaandrew@gmail.com; 5 Department of Pharmacy, School of Health Sciences, Levy Mwanawasa Medical University, Lusaka 10101, Zambia; jimmyhangoma0282@gmail.com; 6 Department of Biomedical Sciences, School of Veterinary Medicine, University of Zambia, Lusaka 10101, Zambia; chikchile@gmail.com (C.C.); ntombi.nkonde@unza.zm (N.B.M.); 7 Ministry of Fisheries and Livestock, Lusaka 10101, Zambia; taonasinyawa@gmail.com (T.S.);; 8 Department of Disease Control, School of Veterinary Medicine, University of Zambia, Lusaka 10101, Zambia; 9 Center for Infectious Diseases Research in Zambia, Lusaka 10101, Zambia; amon.siame@cidrz.org (A.S.); mwendalubi.hadunka@cidrz.org (M.H.); fred.simwinji@cidrz.org (F.M.S.); kaunda.kaunda@cidrz.org (K.K.);; 10 Word Health Organization, Lusaka 10101, Zambia; mchibi@who.int (M.C.); plisulo@gmail.com (P.L.); masaningaf@who.int (F.M.); 11 Zambia Medicines Regulatory Authority, Lusaka 10101, Zambia; jeree@zamra.co.zm (E.J.); msiyanga@zamra.co.zm (M.S.); 12 Hokudai Center for Zoonosis Control in Zambia, Hokkaido University, Lusaka 10101, Zambia; misheckshawa@czc.hokudai.ac.jp; 13 Division of International Research Promotion, International Institute for Zoonosis Control, Hokkaido University, Sapporo 060-0808, Japan; 14 School of Agriculture, University of Zambia, Lusaka 10101, Zambia; mercy.mukuma@unza.zm; 15 Food and Agriculture Organization, Lusaka 10101, Zambia; 16 Division of Research Support, Institute for Vaccine Research and Development, Hokkaido University, Sapporo 001-0021, Japan

**Keywords:** antimicrobial consumption, antimicrobial use, antimicrobial resistance, antimicrobial stewardship, One Health, Zambia

## Abstract

**Background/Objectives:** Antimicrobial Resistance (AMR) remains a growing public health threat, underscoring the need for robust surveillance of Antimicrobial Consumption (AMC) and Antimicrobial Use (AMU). This study analysed AMC and AMU trends in Zambia’s human and animal health sectors, identifying priority areas for antimicrobial stewardship (AMS) under a One Health framework. **Methods:** A retrospective study was conducted in February 2025, utilising data from 2018 to 2023. Sources of data included the Zambia Medicines Regulatory Authority (ZAMRA) and the WOAH Animal Antimicrobial Use (ANIMUSE) Global Database platform. AMC was analysed using the WHO Global Antimicrobial Resistance and Use Surveillance System (GLASS) methodology. Antimicrobials were classified using the WHO Anatomical Therapeutic Chemical (ATC) system, and consumption was measured in Defined Daily Doses (DDDs) per 1000 inhabitants per day (DID). Antibiotics were further categorised using the WHO Access, Watch, and Reserve (AwaRe) classification. Data analysis was performed using IBM SPPS version 25.0. **Results:** In the human health sector, oral antibiotics accounted for 88% of total consumption. Penicillins (33%), cephalosporins (19.2%), and macrolides (12.4%) were the most consumed classes. In 2023, 98,651,882.42 DDDs per 1000 inhabitants/day were recorded, with amoxicillin, ceftriaxone, and sulfamethoxazole/trimethoprim leading as the most consumed antibiotics. According to the consumption of antibiotics by the WHO AwaRe classification, 47% were Access, 40% Watch, and 3% Reserve group antibiotics. In animal health, tetracyclines dominated (63%), followed by sulphonamides (26%) and penicillins (11%). AMU in animal health peaked in 2023. **Conclusions:** This study found high AMC and AMU, especially Watch-group antibiotics and tetracyclines, highlighting the need for strengthened antimicrobial stewardship, regulatory oversight, and integrated One Health surveillance to mitigate AMR risks in Zambia.

## 1. Introduction

The increasing global threat of Antimicrobial Resistance (AMR) has prompted the call for coordinated action across the human, animal and environmental health sectors [1,2,3,4]. AMR occurs when microorganisms stop responding to treatment with antimicrobials [5,6,7,8]. This phenomenon arises primarily from the inappropriate use of antimicrobials, leading to reduced effectiveness of treatments and heightened risks of morbidity, mortality, and economic burdens [6,9,10]. The World Health Organisation (WHO), the Food and Agriculture Organisation (FAO), and the World Organisation for Animal Health (WOAH) have emphasised the need for comprehensive surveillance of Antimicrobial Consumption (AMC) and utilisation patterns to inform policy interventions and mitigate the spread of resistant pathogens [11,12,13,14]. Additionally, there are concerns of increasing rates of AMC, which calls for attention as this could contribute to a rise in AMR [15,16,17].

In human health, antimicrobials are commonly prescribed for bacterial infections, yet empirical prescribing practices, over-the-counter access, and self-medication contribute to excessive consumption [10,18,19,20]. Previous studies in sub-Saharan Africa have demonstrated that a significant proportion of antibiotic prescriptions are unnecessary or inappropriate, highlighting the need for improved regulatory oversight and prescriber education [18,21,22,23]. A recent systematic review and meta-analysis of point prevalence surveys (PPS) across African health facilities reported a pooled antimicrobial prescription rate of 60%, with substantial variation in prescription quality and guideline compliance, underscoring the need for strengthened antimicrobial stewardship (AMS) and surveillance systems across the continent [24].

Globally, there has been an increase in the use of antimicrobials in food-producing animals [25,26,27]. Antibiotics are frequently administered to prevent and treat infections in poultry, cattle, and fish farming, often without veterinary oversight [28,29,30]. The livestock and aquaculture industries also represent key contributors to antimicrobial use (AMU) in Zambia [31,32,33,34]. The use of antimicrobials as growth promoters, a practice discouraged by global health agencies, further exacerbates the risk of AMR transmission from animals to humans through the food chain and the environment [35,36]. Evidence has shown that tetracyclines, sulphonamides, quinolones, and penicillins are highly used in the animal health sector [37,38]. A clearer understanding of AMU patterns in animal health is necessary to promote responsible antimicrobial use while sustaining agricultural production and productivity [39,40].

Several strategies have been instigated to address the overconsumption and misuse of antimicrobials across the One Health sectors globally [20,27,41,42,43]. Countries have strengthened monitoring of AMU through periodic surveys, including point-prevalence surveys (PPS) and prescription audits [44,45]. Alongside this, emphasis has been made on the need for healthcare workers to adhere to global, national, and local treatment guidelines [46,47,48]. Among the available guidelines that promote the rational use of antibiotics are the WHO Access, Watch, and Reserve group antibiotics [49,50,51]. The Access group antibiotics are recommended as first- or second-choice treatments for common infectious syndromes due to their proven efficacy, safety, and lower potential to drive AMR [52,53,54]. The Watch group antibiotics have a higher resistance potential and are prioritised as key targets for stewardship interventions because their overuse may accelerate the development of AMR [52,53,55]. The Reserve group antibiotics are designated as last-resort options for treating confirmed or suspected infections caused by multidrug-resistant (MDR) organisms, and their use should be restricted to preserve their effectiveness for critical clinical situations [52,56]. Additionally, rational use of antibiotics is promoted through the development and enhancement of AMS programmes [57,58,59,60,61,62]. Similarly, there is a need to reduce AMU in food-producing animals through global strategies, including implementing enforceable international regulations that capture AMU, promoting adherence to nutritional guidelines that reduce meat consumption, and introducing a global user fee on veterinary antimicrobials [63].

Zambia, like many low- and middle-income countries (LMICs), faces significant challenges in addressing AMR due to weak regulation and enforcement of AMU, limited access to quality medicines, and critical gaps in surveillance data [64,65,66]. Both the human and animal health sectors contribute to AMU, with antibiotics widely used for therapeutic, prophylactic, and growth-promoting purposes [67,68,69,70]. In the absence of stringent monitoring mechanisms, the indiscriminate use of antimicrobials in livestock production and healthcare settings may accelerate the emergence of resistant bacterial strains, compromising treatment outcomes and food security [71,72]. Many efforts have been implemented to address AMR in Zambia through improved surveillance [73,74,75,76,77]. Through the implementation of the National Action Plan (NAP) on AMR and the integrated AMR surveillance framework, Zambia has made progress in the fight against AMR [78,79]. The main objective of this study was to evaluate AMC in humans and AMU in the animal health sector in Zambia to identify the most consumed antimicrobials and classify them by the WHO AWaRe classification, with the overarching goal of reducing AMC and optimising AMU, thereby minimising the emergence and spread of AMR.

## 2. Results

### 2.1. Human Health AMC Findings

**Figure 1:**
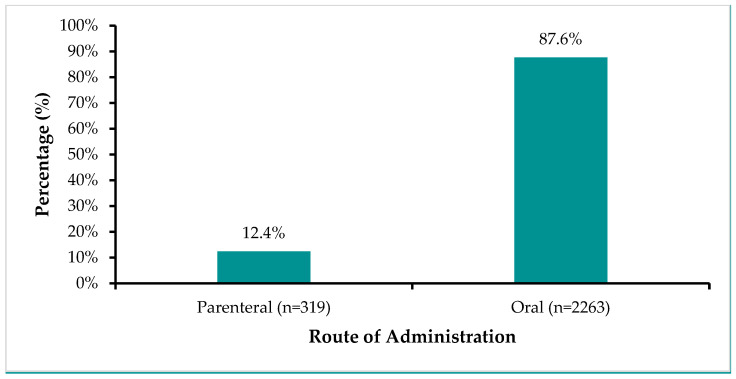
Frequency and Percentage of Antibiotics by Formulation Type, 2023.

Out of 2294 antimicrobials found on the ZAMRA database in 2023, the most consumed antibiotics by route of administration were oral formulations at 2263 (87.6%), as shown in Figure 1.

**Figure 2:**
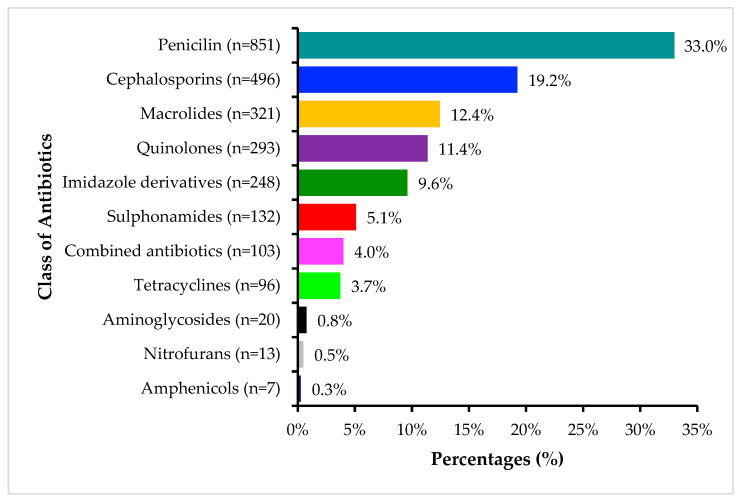
Most Consumed Classes of Antibiotics in the Human Health Sector in Zambia, 2023.

Figure 2 shows that out of the 2582 consumed antimicrobials, penicillins were the most used class at 851 (33.0%), followed by cephalosporins at 496 (19.2%), macrolides at 321 (12.4%), and quinolones at 293 (11.4%). The least consumed antimicrobials were amphenicols at 7 (0.3%).

**Figure 3:**
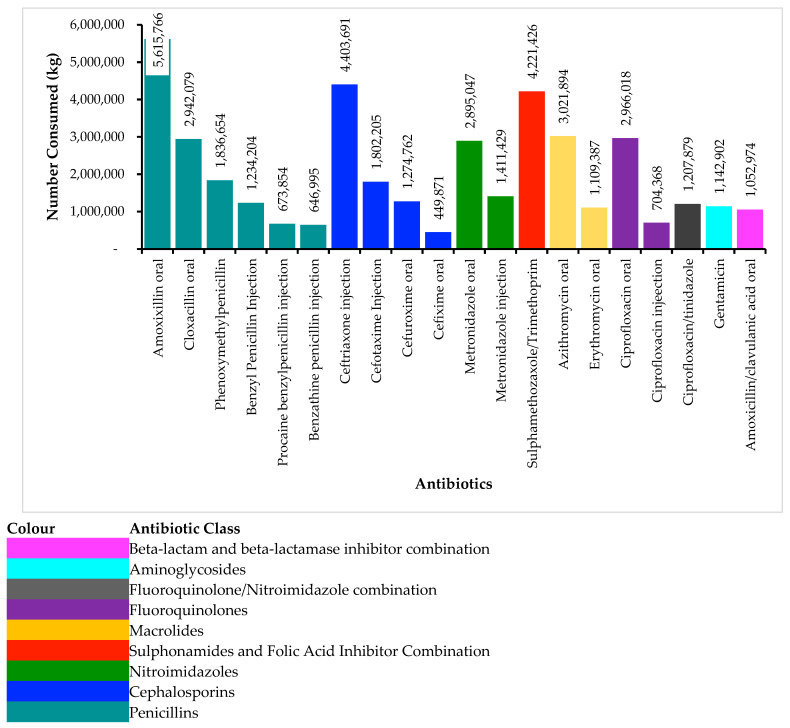
The top 20 antibiotics, colour-coded by class, that were consumed in human health in Zambia in 2023.

Evaluation of the top 20 antibiotics grouped into classes that were consumed in human health in Zambia in 2023 (as shown in Figure 3) revealed a total consumption of 40,613,405 antibiotics. Those that ranked the highest included amoxicillin oral 5,615,766 (12.9%), ceftriaxone 4,403,691 (10.12%), sulphamethoxazole/trimethoprim oral 4,221,426 (9.7%), azithromycin oral 3,021,894 (6.94%), ciprofloxacin oral 2,966,018 (6.82%), cloxacillin oral 2,942,079 (6.76%), and metronidazole oral 2,989,547 (6.65%) (Figure 3).

The total defined daily doses (DDDs) of antibiotics consumed in Zambia in 2023 amounted to 98,651,882.42, with a Defined Daily Dose per 1000 inhabitants per day (DID) of 13.78. The most consumed individual antibiotic was oral amoxicillin, contributing 8.4 million DDDs (12.91%), followed closely by ceftriaxone injection with 8.8 million DDDs (10.12%), and sulfamethoxazole/trimethoprim oral with 33.7 million DDDs (9.7%). Other frequently used antibiotics included ciprofloxacin oral (6.82%), azithromycin oral (6.94%), cloxacillin oral (6.76%), and oral metronidazole (6.65%) (Table 1).

In terms of the WHO AwaRe classification, Access group antibiotics dominated, accounting for over 46.8% of total consumption, with agents like amoxicillin, metronidazole, cloxacillin, and sulfamethoxazole/trimethoprim leading. Watch group antibiotics, such as ceftriaxone, cefotaxime, azithromycin, ciprofloxacin, and levofloxacin, made up a significant portion of the remaining consumption (40.3%), while Reserve group antibiotics, comprising linezolid and daptomycin, were minimally used (3.2%), reflecting appropriate stewardship in terms of reserving last-line therapies (Table 1).

**Figure 4:**
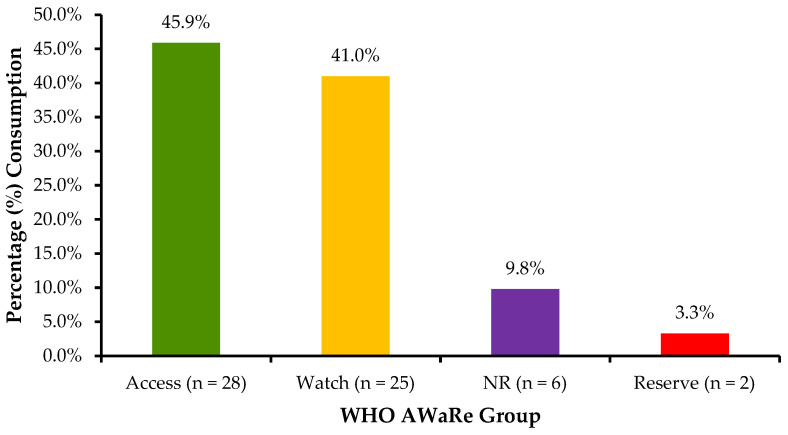
Consumption of antibiotics based on the WHO AwaRe classification.

Figure 4 shows the proportion of antibiotics categorised by the WHO AwaRe classification framework. The results indicate that a total of 61 antibiotics were consumed in 2023, with the majority being ‘Access’ at 28 (45.9%) and the least being ‘Reserve’ at 2 (3.3%).

### 2.2. Animal Health AMU Findings

**Figure 5:**
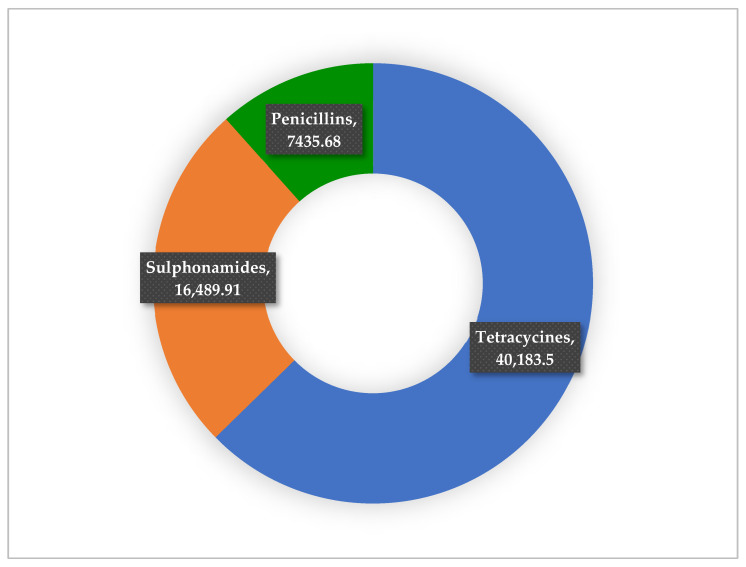
Shows the percentages of the most used antibiotics in animal health.

The data shows that tetracycline use was the highest at 40,183.5 kg (63%), followed by sulphonamides at 16,489.91 kg (26%), with the least being penicillins at 7435.68 kg (11%) (Figure 5).

**Figure 6:**
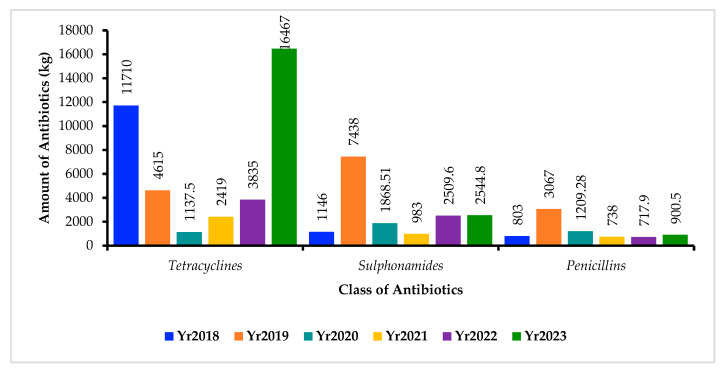
Shows the Most Common Antibiotics Consumed (in Kilograms) in Animal Health between 2018 and 2023. Note: Yr = Year.

When split by year, the data shows that in 2018, tetracycline was the highest at 11,710 kg, followed by sulphonamides at 1146 kg, with the least being penicillin at 803 kg. In 2019, sulphonamides were the highest at 7438 kg, tetracycline at 4614 kg and penicillin at 3067 kg. In 2020, sulphonamide use was at 1868.51 kg, penicillin at 1209.28 kg, and tetracycline was the least used at 1137.5 kg. In 2021, tetracycline had the highest use at 2419 kg, sulphonamides at 983 kg and penicillin at 738 kg. In 2022, the use of tetracyclines was the highest at 3835 kg, followed by sulphonamides at 2509.6 kg, and penicillin at 717.9 kg. In 2023, tetracyclines were the highest at 16,467 kg, followed by sulphonamides at 2544.8 kg and penicillin at 900.5 kg. In the period between 2018 and 2023, 2023 had the highest use of antibiotics at 19,912.3 kg, followed by 2019 at 15,120 kg. The least use of antibiotics was seen in 2022 at a total of 4140 kg (Figure 6).

## 3. Discussion

To the best of our knowledge, this is the first national study on AMC and AMU across the human and animal health sectors in Zambia. The study found that the most consumed classes in the human health sector were oral antibiotics, which accounted for the majority of use, followed by penicillins, cephalosporins, and macrolides. It also established that the most frequently used antibiotics were amoxicillin, ceftriaxone, and sulfamethoxazole/trimethoprim. According to the WHO AwaRe classification, antibiotic use was predominantly in the Access category, followed closely by Watch antibiotics, with a small proportion classified as Reserve. In the animal health sector, tetracyclines were the most used class, followed by sulphonamides and penicillins. Total antimicrobial consumption reached its highest level in 2023. These findings highlight the need for continuous monitoring of AMC and AMU in both the human and animal health sectors in Zambia.

Our study found that oral formulations consistently accounted for 87.6% of total antimicrobial consumption each year, highlighting a strong and sustained preference for oral administration in Zambia. This trend aligns with findings from African countries where AMC of oral antibiotics was 97% in Sierra Leone from 2017 to 2019 [80] and exceeded 87% in Ethiopia between 2020 and 2022 [16], respectively. Additionally, these results are consistent with studies in Uganda, where oral antimicrobials constituted 80.7% of total consumption, and 86% of products supplied by the Joint Medical Store (JMS) were oral formulations [81,82]. Comparable trends were observed in the WHO European and ESAC-Net countries, where oral amoxicillin/clavulanic acid ranked as the most consumed antibiotic in 52% and 33% of countries, respectively, while oral amoxicillin ranked second overall [83]. Japan also reported a dominant reliance on oral formulations, accounting for 93% of total AMU over 13 years [84]. The preference for oral administration is likely attributed to its convenience, affordability, and ease of use, especially in outpatient settings [85]. However, this widespread use also raises concerns about inappropriate antimicrobial use and the potential for resistance development, particularly in settings with limited diagnostic capacity and over-the-counter antibiotic access. Therefore, targeted interventions such as stricter regulation, public education, and improved access to diagnostics are essential to optimise oral antibiotic use and mitigate the risk of antimicrobial resistance.

In this study, the most widely consumed classes of antibiotics in Zambia were penicillins (33%), cephalosporins (19.2%), macrolides (12.4%), quinolones (11.4%), and imidazoles (9.6%). This pattern reflects a global reliance on broad-spectrum and commonly prescribed antimicrobials in clinical settings. The high use of penicillins aligns with findings from Uganda, where penicillins consistently accounted for the largest share of AMC over three years—41% in 2017, 42% in 2018, and 36% in 2019 [81]. Similar trends were observed in the WHO European and ESAC-Net countries, where penicillins also ranked among the most commonly used classes, followed by tetracyclines, cephalosporins, and quinolones [83]. However, some regional variations are evident. In Ethiopia, tetracyclines (35.81%) were the most consumed class, followed by fluoroquinolones (20.19%), macrolides (13.92%), antiretrovirals (10.57%), and cephalosporins (9.63%) [86]. In contrast, a study in Japan identified macrolides (32%) and cephalosporins (28%) as the dominant classes, with fluoroquinolones also ranking highly at 19% [84]. These variations may reflect differences in disease epidemiology, prescribing habits, availability, and regulatory environments. The high consumption of cephalosporins and quinolones in Zambia is noteworthy, given their classification in the WHO AwaRe framework as Watch group antibiotics, agents that should be used with caution due to their higher potential for driving AMR. These findings highlight the need to strengthen AMS programmes and promote the rational use of antibiotics, particularly those in the Watch group, to mitigate AMR risks in Zambia.

The present study found that the most frequently consumed antimicrobials in Zambia in 2023 were amoxicillin (oral) at 5,615,766 units (12.9%), followed by ceftriaxone (4,403,691; 10.1%), sulfamethoxazole/trimethoprim (4,221,426; 9.7%), azithromycin (3,021,894; 6.9%), and ciprofloxacin (2,966,018; 6.8%). Other notable antibiotics included cloxacillin (6.8%), metronidazole (oral and injectable, 6.7% and 3.2%, respectively), phenoxymethyl penicillin (4.2%), and cefotaxime injection (4.1%). These findings mirror consumption patterns observed in several LMICs, where broad-spectrum antibiotics such as amoxicillin and ceftriaxone remain the mainstay of empirical therapy. The high usage of amoxicillin aligns with studies from Uganda and across multiple countries, where it ranked as the most consumed antibiotic in both oral form and in combination with clavulanic acid [81,83,87,88]. Similarly, the widespread use of sulfamethoxazole/trimethoprim and azithromycin has been reported in East African settings, often attributed to their availability, affordability, and inclusion in national treatment guidelines [81]. However, the substantial consumption of ceftriaxone and other parenteral antibiotics such as cefotaxime and benzylpenicillin raises concerns about potential overuse of Watch group antibiotics, especially in outpatient or empiric settings. The high use of ceftriaxone has been reported in other LMIC settings, raising concerns of misuse and potential for the development and spread of highly resistant pathogens against this Watch group antibiotic [76,77,89,90,91,92,93,94,95]. The high use of ceftriaxone has been reported in previous studies in Zambia, which raises a lot of concerns [75,76,77,96,97,98,99]. Ceftriaxone, a WHO highest-priority critically important antimicrobial, is being overused in Zambia, driving the rise in ESBL-producing pathogens. Without stronger stewardship and regulation, this misuse threatens to undermine one of the few effective treatments for severe infections. On the other hand, a study conducted in Japan reported that clarithromycin and ceftriaxone were the top used antibiotics, reflecting variations in prescribing practices and regulatory environments [84], while Tanzania showed a slightly different trend with doxycycline and amoxicillin leading [88]. These differences likely reflect variations in prescribing practices, diagnostic capacity, and national formulary preferences. The predominance of oral antibiotics observed in our study underscores the importance of monitoring AMC trends to inform stewardship interventions and minimise the risk of resistance development.

In this study, a total of 98.7 million DDDs of antibiotics were consumed in Zambia’s human health sector in 2023, yielding a Defined Daily Dose per 1000 inhabitants per day (DID) of 13.78. The most frequently consumed antibiotics were oral amoxicillin (12.91%), ceftriaxone injection (10.12%), and oral sulfamethoxazole/trimethoprim (9.7%), followed by other commonly used agents such as azithromycin, ciprofloxacin, cloxacillin, and oral metronidazole. These findings are consistent with previous studies from the region, including those by Namugambe et al. (2021) in Uganda, where oral amoxicillin and sulfamethoxazole/trimethoprim were among the most widely used antibiotics [81]. Similarly, Robertson et al. (2021) identified oral amoxicillin as the top-ranked antibiotic in six countries and second overall across two surveillance networks, highlighting its widespread use as a first-line agent [83]. The high consumption of ceftriaxone, a parenteral β-lactam antibiotic, aligns with findings from both Uganda and India, where it was also the most consumed injectable antibiotic [81,100].

Using the WHO AWaRe classification, Access group antibiotics accounted for approximately 46% of total consumption, including high-use agents like amoxicillin, cloxacillin, and metronidazole. Watch group antibiotics such as ceftriaxone, azithromycin, and ciprofloxacin comprised approximately 40%, while Reserve antibiotics such as linezolid and daptomycin were used minimally (3.3%), indicating appropriate stewardship of last-resort therapies. These findings are in line with trends observed in other LMICs, including Ethiopia, Uganda, Tanzania, and Sierra Leone, where Access group antibiotics comprised the majority of consumption [16,80,81,82,88]. The presence of non-recommended (NR) fixed-dose combinations (9.8%) also warrants further regulatory attention to ensure rational use of antimicrobials and alignment with WHO guidelines. Despite Access group antibiotics being the most consumed antibiotics, the rate of consumption is lower than that recommended by the WHO, which was initially set at a 60% threshold [52,53,55,56], but now stands at 70% [101]. The challenge of failing to meet the earlier 60% and currently 70% threshold of using Access group antibiotics has been reported in other studies. This highlights the high use of Watch group antibiotics, indicating deviations from the treatment guidelines and protocols [102,103,104,105,106]. Therefore, there is a need to strengthen stewardship interventions to promote the rational use of antibiotics and reduce the overuse of Watch group antibacterials.

The present study revealed considerable year-to-year variation in AMU within the veterinary sector in Zambia between 2018 and 2023. Overall, tetracyclines emerged as the most consumed class, accounting for 63% (40,183.5 kg) of total usage, followed by sulphonamides (26%) and penicillins (11%). Similarly, local sales data reported by other authors in Zambia showed high consumption of tetracyclines and sulphonamides [34,107,108,109]. The consumption pattern has been reported in the sub-Sharan African region [37]. This trend mirrors global patterns where tetracyclines are frequently used in animal health due to their broad-spectrum activity, affordability, and ease of access [17]. The high consumption of medically important antibiotics in animal health has also been reported in other countries [110]. Yearly disaggregation showed that tetracyclines dominated usage in 2018 (11,710 kg), dropped sharply in 2020 (1137.5 kg), but rebounded significantly to reach a peak in 2023 (16,467 kg). Conversely, sulphonamides peaked in 2019 (7438 kg), followed by a decline and then a modest increase in 2023 (2544.8 kg). Penicillin usage showed a gradual rise from 803 kg in 2018 to 3067 kg in 2019, before declining steadily through 2022 (717.9 kg), with a slight rebound in 2023 (900.5 kg). These fluctuations may reflect variations in disease outbreaks, shifts in treatment protocols, or supply chain dynamics. The highest total antibiotic use was recorded in 2023 (19,912.3 kg), possibly influenced by increased livestock production or relaxed restrictions post-COVID-19, whereas 2022 saw the lowest usage (4140 kg), which may indicate improved AMS efforts or reduced demand. Similar year-to-year variations in veterinary antibiotic use have been documented elsewhere, reinforcing the need for robust AMU surveillance systems [19,111,112]. The increase in global AMC and AMU in animal health has been reported in other studies and is among the major causes of AMR [17,111,113,114,115,116]. The high demand for animal proteins, especially in LMICs, has contributed to the increased AMU, which is also linked to the development and spread of AMR [113]. These trends underscore the importance of national strategies aimed at rationalising antibiotic use in animal health, especially in the context of Zambia’s One Health response to AMR.

Overall, these results highlight critical trends in AMC and AMU in Zambia and reinforce the importance of strengthening surveillance and AMS programmes, developing treatment guidelines in animal health and updating national treatment guidelines in human health, and integrating the WHO AWaRe categorisation into procurement and prescribing policies to combat the growing threat of AMR. The study highlights high AMC and AMU in Zambia, which, when connected to animal health, reflects weak veterinary stewardship programmes and gaps in enforcing prescription-only regulations, record-keeping, and quality control of veterinary medicines. These regulatory shortcomings contribute to inappropriate antimicrobial use in livestock, amplifying the risk of resistance transmission across human, animal, and environmental sectors.

Our findings underscore the need for multifaceted One Health strategies to address the high levels of AMC and AMU observed across both the human and animal health sectors. Such strategies should not only focus on reducing inappropriate prescribing and dispensing practices in healthcare settings but also on strengthening regulatory frameworks to curb over-the-counter sales and unsupervised use in the community [44,117]. In the animal health sector, targeted interventions are required to promote prudent veterinary oversight, restrict non-therapeutic use of antimicrobials such as growth promotion, and encourage alternative disease prevention methods, including vaccination, biosecurity, and improved husbandry practices [20,118,119,120]. Furthermore, investment in diagnostic capacity, surveillance systems, and AMS programmes is essential to generate high-quality evidence that informs policy and guides rational AMU [121,122,123]. These actions align with global and regional recommendations and are critical to mitigating the risk of AMR while safeguarding the efficacy of existing drugs for both human and animal health. The policy recommendations and implications of the present study findings are outlined in Table 2.

We are aware that this study has some limitations. First, as in many LMICs, antimicrobials in Zambia are frequently sold over the counter without prescriptions, making it difficult to capture the actual quantities used and the specific populations treated. Second, farm-level data were limited by poor record-keeping, particularly among smallholder farmers, which may have obscured detailed usage patterns. Third, the lack of veterinary oversight in many rural areas restricted the ability to monitor antimicrobial use comprehensively in the animal health sector. Fourth, the study relied primarily on importation and sales data as proxies for consumption, which may not fully reflect actual end-user use due to possible stockpiling, wastage, or informal distribution channels. Finally, the analysis did not capture environmental antimicrobial residues or their contribution to AMR, an area warranting further investigation.

Future studies on AMC and AMU in Zambia should focus on bridging data and practice gaps. Community- and hospital-based surveys are needed to capture actual prescribing, dispensing, and self-medication patterns. At the farm level, detailed investigations should assess antimicrobial use among commercial and smallholder farmers and its link to resistance in humans, food, and the environment. Environmental surveillance should examine residues and resistant organisms in water, soil, and waste streams.

Research should also evaluate how limited diagnostic capacity affects prescribing and explore low-cost diagnostic innovations. Studies assessing the effectiveness of AMS interventions, including digital tools, treatment guidelines, and training, are vital for both human and animal health sectors. Integrated One Health surveillance linking human, animal, and environmental data remains a priority.

Further, policy-focused research should assess the impact of regulations on prescribing and veterinary drug sales. Investigating alternatives to antimicrobials, such as vaccines, probiotics, and biosecurity measures, will provide sustainable solutions. Behavioural and cultural studies can illuminate factors driving antibiotic misuse, while economic evaluations should quantify the burden of AMR and cost-effectiveness of AMS interventions.

## 4. Materials and Methods

### 4.1. Study Setting and Data Sources

This was a retrospective study that was conducted in December 2024, utilising routine data collected between 2018 and 2023 in the human and animal health sectors. The routine AMC and AMU data were obtained from human and animal health databases in Zambia.

### 4.2. Antimicrobial Consumption (AMC) in Human Health in Zambia

Zambia, a landlocked nation in Southern Africa, has a pharmaceutical sector that includes both public and private providers. The majority of medicines used in the country are imported from India, Kenya, the Netherlands, South Africa, and China, with only 10% to 15% of pharmaceutical production coming from local manufacturers [124]. The Zambia Medicines Regulatory Authority (ZAMRA) is responsible for regulating the pharmaceutical sector, including granting marketing authorisations, ensuring compliance with good manufacturing practices, licencing pharmaceutical outlets, and overseeing import and export activities [125].

The approval process for pharmaceutical products is managed by technical committees specialising in human and veterinary medicines. To strengthen regulatory oversight, ZAMRA implemented the Integrated Regulatory Information Management System (IRIMIS), an online platform designed to systematically collect data on all imported consignments of finished pharmaceutical products and raw materials, including antimicrobials. This system enables national-level monitoring of medicine consumption, ensuring alignment with long-term health priorities. Additionally, IRIMIS has digitised import data, replacing the previous paper-based system. Historical records were retrieved and entered into the system, which captures key data on antimicrobial imports and exports at designated ports of entry.

The primary data sources for this report included ZAMRA’s import permits, proforma and commercial invoices, based on the data that is submitted to the WHO Global Antimicrobial Resistance and Use Surveillance System (GLASS) methodology. Human health AMC data were collected for 2023.

All import data on antimicrobials were included in the analysis as they serve as a proxy for overall national consumption, encompassing both the public and private sectors, given that ZAMRA authorises all imports regardless of their final distribution. The key variables assessed included product details (name, active ingredient(s), strength, dosage form, and route of administration), batch size, quantity, unit cost, WHO ATC classification, WHO AWaRe classification of antibiotics, and DDD. The WHO AWaRe classifications of antibiotics were performed based on the recommended guidelines [49,51]. The extracted data from IRIMIS was compiled into Microsoft Excel^®^ for further analysis.

### 4.3. Antimicrobial Use (AMU) in Animal Health in Zambia

Animal health data were sourced from the WOAH Animal Antimicrobial Use (ANIMUSE) Global Database, managed by the World Organisation for Animal Health (WOAH). This dataset includes AMU data submitted by Zambia to the WHO’s Global Antimicrobial Resistance and Use Surveillance System (GLASS) and WOAH from 2018 to 2023. Additionally, the report incorporates defined daily dose (DDD) data for animal health to evaluate trends over time.

### 4.4. Animal Health Data Collection

Animal health data were collected and analysed using the ANIMUSE Global Database, a platform managed by WOAH that facilitates access to critical and expanding information on AMU in animals [126]. This database compiles country-level data on antimicrobial quantities and usage patterns in animal health. Zambia has been submitting AMC data to ANIMUSE since 2018, and this report reviews data from 2018 to 2023. Antibiotics were classified by their classes.

### 4.5. Data Analysis

The collected data were entered into a Microsoft Excel sheet and checked for completeness and duplication. Data analysis was conducted using IBM SPSS (Version 25.0; IBM Corp., Armonk, NY, USA). The analysis of AMC and AMU focused on calculating and visualising key indicators. At the national level, antibiotic quantities were determined using DDD and DID calculations [127,128,129]. The analysis also identified the 20 most frequently consumed antibiotic substances. Additionally, the distribution of AMC was examined using the Drug Utilisation 75% (DU75) and 90% (DU90) metrics to highlight the most commonly used antibiotics [130]. Data were further stratified based on the route of administration to better understand usage patterns across different delivery methods, similar to previous studies [81,82].

## 5. Conclusions

This study provides the first comprehensive national analysis of AMC and AMU in both the human and animal health sectors in Zambia, offering critical insights for optimising antimicrobial stewardship across the One Health spectrum. The findings reveal a high reliance on oral formulations in human health and a predominance of penicillins, cephalosporins, and macrolides, many of which fall under the WHO Watch category, signalling the need for cautious use. The high utilisation of tetracyclines and sulphonamides in animal health further underscores the importance of prudent AMU in veterinary settings to curb the emergence and spread of AMR.

The substantial consumption of broad-spectrum and Watch group antibiotics in human health, along with year-to-year variability in veterinary AMU, highlights gaps in AMS, surveillance infrastructure, and regulatory enforcement. Importantly, the observed use of non-recommended (NR) antibiotic combinations calls for strengthened regulatory oversight and alignment with WHO guidelines.

To mitigate AMR threats, Zambia must prioritise the integration of the WHO AWaRe classification into procurement and prescribing policies, enhance diagnostic capacity to support rational antibiotic use, and expand the coverage and quality of AMU surveillance. Additionally, scaling up multisectoral collaboration, investing in stewardship training, research, and enforcing national treatment guidelines are critical steps toward safeguarding antimicrobial efficacy.

Overall, this study establishes a foundation for evidence-based policy development and targeted interventions to ensure the sustainable use of antimicrobials in Zambia, in alignment with global One Health strategies to combat AMR.

## Figures and Tables

**Table 1:** Antibiotic consumption in human health in Zambia, 2023.

No.	Medicine Name	ATC5	Class	Formulation	Total Package	DDD_WHO	Total DDD	DID	%	AwaRe Class
1	Doxycycline oral	J01AA02	Tetracycline	Oral	289,733	0.1	28,973.30	0.00405	0.67	Access
2	Minocycline oral	J01AA08	Tetracycline	Oral	200	0.2	40.00	0.00001	0	Watch
3	Tetracycline oral	J01AA07	Tetracycline	Oral	218,862	1	218,862.00	0.03058	0.5	Access
4	Chloramphenicol injection	J01BA01	Amphenicol	Parenteral	30	3	90.00	0.00001	0	Access
5	Chloramphenicol oral	J01BA01	Amphenicol	Oral	72,910	3	218,730.00	0.03056	0.17	Access
6	Amoxicillin oral	J01CA04	Penicillin	Oral	5,615,766	1.5	8,423,649.00	1.17683	12.91	Access
7	Ampicillin oral	J01CA01	Penicillin	Oral	1349	2	2698.00	0.00038	0	Access
8	Benzathine Penicillin injection	J01CE08	Penicillin	Parenteral	646,995	3.6	2,329,182.00	0.32540	1.49	Access
9	Benzylpenicillin injection	J01CE01	Penicillin	Parenteral	1,234,204	3.6	4,443,134.40	0.62073	2.84	Access
10	Clavulanate/amoxicillin injection	J01CC02	Penicillin	Parenteral	1042	3	3126.00	0.00044	0	Access
11	Clavulanate/amoxicillin oral	J01CC02	Penicillin	Oral	1,052,974	1.5	1,579,461.00	0.22066	2.42	Access
12	Cloxacillin injection	J01CF02	Penicillin	Parenteral	56,886	2	113,772.00	0.01589	0.13	Access
13	Cloxacillin oral	J01CF02	Penicillin	Oral	2,942,079	2	5,884,158.00	0.82205	6.76	Access
14	Cloxacillin/Ampicillin oral	J01CA51	Penicillin	Oral	180,379	4	721,516.00	0.10080	0.41	Access
15	Flucloxacillin oral	J01CF05	Penicillin	Oral	23,530	2	47,060.00	0.00657	0.05	Access
16	Phenoxymethyl penicillin	J01CE02	Penicillin	Oral	1,836,654	2	3,673,308.00	0.51318	4.22	Access
17	Piperacillin/Tazobactam injection	J01CR05	Penicillin	Parenteral	14,182	14	198,548.00	0.02774	0.03	Watch
18	Procaine Benzyl Penicillin injection	J01CE09	Penicillin	Parenteral	673,854	0.6	404,312.40	0.05648	1.55	Access
19	Sulbactam/Ampicillin injection	J01CR01	Penicillin	Parenteral	8200	12	49,200.00	0.00687	0.02	Access
20	Cefaclor oral	J01DC52	Cephalosporin	Oral	30,000	1	30,000.00	0.00419	0.07	Watch
21	Cefadroxil oral	J01DH51	Cephalosporin	Oral	4	2	8.00	0.00000	0	Access
22	Cefazolin Injection	J01DB04	Cephalosporin	Parenteral	6022	3	18,066.00	0.00252	0.01	Access
23	Cefdinir oral	J01DD15	Cephalosporin	Oral	6430	0.6	3858.00	0.00054	0.01	Watch
24	Cefepime injection	J01DE01	Cephalosporin	Parenteral	84,850	4	339,400.00	0.04742	0.19	Watch
25	Cefixime oral	J01DD08	Cephalosporin	Oral	449,871	0.4	179,948.40	0.02514	1.03	Watch
26	Cefixime/Lactic acid bacillus oral	J01DD08	Cephalosporin	Oral	18,581	0.4	7432.40	0.00104	0.04	Watch
27	Cefotaxime injection	J01DD01	Cephalosporin	Parenteral	1,802,205	4	7,208,820.00	1.00711	4.14	Watch
28	Cefoxitin Sodium Injection	J01DC01	Cephalosporin	Parenteral	2	6	12.00	0.00000	0	Watch
29	Cefpodoxime oral	J01DD13	Cephalosporin	Oral	118,158	0.4	47,263.20	0.00660	0.27	Watch
30	Ceftriaxone Injection	J01DD04	Cephalosporin	Parenteral	4,403,691	2	8,807,382.00	1.23044	10.12	Watch
31	Cefuroxime oral	J01DC02	Cephalosporin	Oral	1,274,762	0.5	637,381.00	0.08905	2.93	Watch
32	Cilastatin/Imipenem	J01DH51	Carbapenem	Parenteral	75,818	2	151,636.00	0.02118	0.17	Watch
33	Clavulanate/cefuroxime	J01DC52	Cephalosporin	Oral	10	0.5	5.00	0.00000	0	Watch
34	Sulphamethoxazole/Trimethoprim oral	J01EE01	Sulphonamide/Trimethoprim	Oral	422,1426	8	33,771,408.00	4.71804	9.7	Access
35	Azithromycin oral	J01FA10	Macrolide	Oral	3,021,894	0.3	906,568.20	0.12665	6.94	Watch
36	Clarithromycin oral	J01FA09	Macrolide	Oral	136,448	0.5	68,224.00	0.00953	0.31	Watch
37	Clindamycin oral	J01FF01	Lincosamide	Oral	62,147	1.2	74,576.40	0.01042	0.14	Access
38	Roxithromycin oral	J01FA06	Macrolide	Oral	15	0.3	4.50	0.00000	0	Watch
39	Erythromycin oral	J01FA01	Macrolide	Oral	1,109,387	1	1,109,387.00	0.15499	2.55	Watch
40	Amikacin oral	J01GB06	Aminoglycoside	Oral	7406	0.59	4369.54	0.00061	0.02	Access
41	Gentamycin	J01GB03	Aminoglycoside	Parenteral	1,142,902	0.24	274,296.48	0.03832	2.63	Access
42	Kanamycin	J01GA08	Aminoglycoside	Parenteral	100	3	300.00	0.00004	0	Watch
43	Ciprofloxacin injection	J01MA02	Fluoroquinolone	Parenteral	704,368	0.8	563,494.40	0.07872	1.62	Watch
44	Ciprofloxacin oral	J01MA02	Fluoroquinolone	Oral	2,966,018	1	2,966,018.00	0.41437	6.82	Watch
45	Levofloxacin injection	J01MA12	Fluoroquinolone	Parenteral	14,426	0.5	7213.00	0.00101	0.03	Watch
46	Levofloxacin oral	J01MA12	Fluoroquinolone	Oral	323,587	0.5	161,793.50	0.02260	0.74	Watch
47	Moxifloxacin	J01MA14	Fluoroquinolone	Oral	656	0.4	262.40	0.00004	0	Watch
48	Nalidixic oral	J01MB02	Fluoroquinolone	Oral	80,854	4	323,416.00	0.04518	0.19	Access
49	Ciprofloxacin/Tinidazole	J01R	Fluoroquinolone/Imidazole derivative	Oral	1,207,879	2	2,415,758.00	0.33749	2.78	NR
50	Fluconazole/Azithromycin/Secnidazole	J01RA07	Azole antifungal/Macrolide/Nitroimidazole	Oral	42,812	4	171,248.00	0.02392	0.1	NR
51	Metronidazole/Norfloxacin	J01RA14	Imidazole derivative/Fluoroquinolone	Oral	90,783	2	181,566.00	0.02537	0.21	NR
52	Ofloxacin/ornidazole oral	J01RA09	Fluoroquinolone/Nitroimidazole	Oral	406,783	2	813,566.00	0.11366	0.93	NR
53	Tinidazole/Norfloxacin	J01RA13	Imidazole derivative/Fluoroquinolone	Oral	10,000	2	20,000.00	0.00279	0.02	NR
54	Daptomycin injection	J01XX09	Lipopeptide	Parenteral	200	0.28	56.00	0.00001	0	Reserve
55	Linezolid oral	J01XX08	Oxazolidinone	Oral	128,468	1.2	154,161.60	0.02154	0.3	Reserve
56	Metronidazole injection	J01XD01	Imidazole derivative	Parenteral	1,411,429	1.5	2,117,143.50	0.29578	3.24	Access
57	Metronidazole oral	J01XD01	Imidazole derivative	Oral	2,895,047	2	5,790,094.00	0.80891	6.65	Access
58	Metronidazole/Furazolidone oral	J01XB51	Imidazole derivative/Nitrofuran	Oral	155,366	5	776,830.00	0.10853	0.36	NR
59	Tinidazole oral	J01XD02	Imidazole derivative	Oral	1100	2	2200.00	0.00031	0	Access
60	Vancomycin injection	J01XA01	Glycopeptide	Parenteral	89,177	2	178,354.00	0.02492	0.2	Watch
61	Nitrofurantoin oral	P01AE01	Nitrofuran	Oral	142,709	0.2	28,541.80	0.00399	0.33	Access
	TOTAL				43,513,620	136.41	98,651,882.42	13.78218	100	

**Table 2:** Policy Recommendations and Practice Implications for Strengthening AMR Control in Zambia’s Animal and Human Health Sectors (One Health Approach).

Policy Area	Recommendations	Practice Implications
AMR Surveillance and Data Systems	Establish integrated AMR surveillance linking human, animal, and environmental sectors using WHONET and GLASS platforms.	Enables early detection of resistance trends, evidence-based interventions, and global data sharing.
Regulation and Enforcement	Enforce laws regulating antimicrobial production, distribution, and prescription in both human and veterinary sectors.	Reduces inappropriate access and misuse of antimicrobials across all sectors.
Diagnostic and Laboratory Capacity	Strengthen laboratory infrastructure, quality assurance, and capacity for rapid diagnostics in hospitals and veterinary labs.	Improves accurate diagnosis, targeted treatment, and rational antimicrobial use.
Capacity Building and Training	Train healthcare workers, pharmacists, veterinarians, and paraprofessionals on AMR, AMS, and IPC.	Enhances knowledge, improves prescribing/dispensing practices, and promotes infection prevention.
Antimicrobial Stewardship (AMS)	Develop and institutionalise AMS programmes in hospitals, community pharmacies, and veterinary practices.	Optimises antimicrobial use, prevents resistance, and sustains drug effectiveness.
Infection Prevention and Control (IPC)	Implement robust IPC programmes in hospitals, farms, and food production systems.	Reduces infection rates and minimises the need for antimicrobial use.
One Health Collaboration	Strengthen multisectoral coordination among health, veterinary, agriculture, and environmental agencies.	Promotes comprehensive AMR control strategies and resource sharing.
Public Awareness and Community Engagement	Launch national campaigns on AMU/AMR, targeting the public, farmers, and patients.	Increases responsible antimicrobial use and fosters behaviour change.
Research and Innovation	Invest in R&D for new antimicrobials, diagnostics, vaccines, and alternatives (e.g., probiotics, phage therapy).	Provides sustainable solutions and reduces dependency on existing antimicrobials.
Financing and Sustainability	Secure long-term funding for AMR action plans, surveillance, and workforce development.	Ensures continuity and scalability of AMR interventions.

## Data Availability

The data supporting the reported results can be made available on request from the corresponding authors.

## Abbreviations

The following abbreviations are used in this manuscript:

AMC	Antimicrobial Consumption
AMR	Antimicrobial Resistance
AMS	Antimicrobial Stewardship
AMU	Antimicrobial Use
ANIMUSE	Animal Antimicrobial Use
ATC	Anatomical Therapeutic Chemical
AWaRe	Access, Watch, Reserve
DDD	Define Daily Dose
WHO	World Health Organisation
ZAMRA	Zambia Medicines Regulatory Authority
